# Consumption of food groups and associated factors among children aged 6 to 23 months

**DOI:** 10.1590/1984-0462/2022/40/2021080

**Published:** 2022-04-04

**Authors:** Ilanna Mirela Becker Jorge Siqueira, Ana Paula Kulig Godinho, Elaine Cristina Vieira de Oliveira, Fernanda Pons Madruga, Cesar Augusto Taconeli, Claudia Choma Bettega Almeida

**Affiliations:** a Universidade Federal do Paraná, Curitiba, PR, Brazil.

**Keywords:** Complementary feeding, Eating, Feeding behavior, Infant nutrition, Alimentação complementar, Consumo alimentar, Hábitos alimentares, Nutrição infantil

## Abstract

**Objective:**

To assess the consumption of food groups among children aged 6 to 23 months in day care centers and at home and its associated factors.

**Methods:**

This is a population-based cross-sectional study conducted with children from nursery schools in Guaratuba, Paraná. Parents answered a socioeconomic and demographic questionnaire. Food consumption was assessed by directly weighing the meals offered at the day care center. At home, parents filled an estimated food record. The reported foods were classified into seven food groups. Minimum dietary diversity was calculated by the proportion of children who consumed foods from four or more groups. Data were analyzed by simple and multiple logistic regression, presented as odds ratios.

**Results:**

A total of 213 children participated in this study. The average number of food groups consumed was 4.2±1.0 at home and 4.2±1.2 in day care centers. At the day care center, all children consumed grains, roots, and tubers, while at home, this rate was 99.1%. The egg group was the least consumed both at day care (6.6%) and at home (2.8%). At home, more than 60% of children consumed sweets and sugar-sweetened beverages. Children aged 12 to 23 months were more likely to consume milk and dairy products, as well as flesh foods. Higher income was associated with the consumption of legumes, and older maternal age with the consumption of fruits and vegetables.

**Conclusions:**

At home, children had a predominantly dairy-based diet and a high intake of ultra-processed foods. In day care centers, the consumption of healthy foods was higher, indicating the need for families to participate in the formation of healthy eating habits.

## Resumo

**Objetivo:**

Avaliar o consumo de grupos alimentares entre crianças de 6 a 23 meses de idade na creche e no domicílio e seus fatores associados.

**Métodos:**

Estudo transversal de base populacional realizado com crianças de centros municipais de educação infantil de Guaratuba, Paraná. Os pais preencheram um questionário socioeconômico e demográfico. O consumo alimentar foi avaliado pela pesagem direta das refeições ofertadas na creche. No domicílio, um registro alimentar estimado foi preenchido pelos pais. Os alimentos relatados foram classificados em sete grupos alimentares. Diversidade alimentar mínima foi calculada pela proporção de crianças que consumiram quatro ou mais grupos. Os dados foram analisados por regressão logística simples e múltipla, apresentados em razões de chances.

**Resultados:**

Participaram do estudo 213 crianças. A média do consumo de número de grupos alimentares foi de 4,2±1,0 no domicílio e 4,2±1,2 na creche. Na creche, todas as crianças consumiram cereais, raízes e tubérculos, e no domicílio, 99,1%. Na creche (6,6%) e no domicílio (2,8%), o grupo dos ovos foi o menos consumido. Em casa, mais de 60% das crianças consumiram doces e bebidas açucaradas. Crianças entre 12 e 23 meses apresentaram maiores chances de consumir leite e derivados e carnes. Maior renda foi associada ao consumo de leguminosas, e maior idade materna, ao consumo de frutas, legumes e vegetais.

**Conclusões:**

No domicílio, as crianças apresentaram uma alimentação predominantemente láctea e alto consumo de alimentos ultraprocessados. Na creche, o consumo de alimentos saudáveis foi maior, apontando a necessidade da participação da família na formação de hábitos alimentares saudáveis.

## INTRODUCTION

During the first two years of life, healthy eating habits comprise exclusive breastfeeding (EBF) up to six months of life and appropriate complementary feeding from the sixth month, with the offer of other foods besides breast milk, in a varied and balanced diet. From the start, meals must include grains, roots, tubers, legumes, flesh foods, eggs, vegetables, and fruits.^1–4^

Families play a key role in the care of the child’s diet. Fresh and minimally processed foods should be prioritized, and the child should be exposed to a variety of healthy foods available in their region and that are traditionally consumed by the family.^4^ In addition to the home environment, many children under two years of age attend nursery schools (*Centros Municipais de Educação Infantil* — CMEI), where they stay for long periods and have some of their daily meals. Therefore, both the home and school environments should provide a diet that contributes to their adequate growth and development, as well as the formation of healthy eating habits.^5^

However, in Brazil, children’s diet is marked by low micronutrient consumption and high energy intake. This scenario reflects the low diet quality in childhood, represented by the wrong introduction of complementary feeding and the excessive consumption of ultra-processed foods (UPF).^6^ The early introduction of these foods compromises short- and long-term health, favoring the development of chronic non-communicable diseases, such as hypertension, diabetes, obesity, and dyslipidemias. Studies show the increasing prevalence of UPF consumption with a high energy contribution and the lower intake of fresh and minimally processed foods.^7,8,9,10,11^

The importance of the diet in childhood and the role of home and school environments in the child’s health, as well as in the formation of their eating habits, make it imperative to know the food consumption in this age group so as to prioritize actions that promote health and adequate nutrition, improving the current scenario.^1,2^ Considering the arguments above, the present study aimed to evaluate the consumption of food groups among children aged 6 to 23 months and its associated factors.

## METHOD

This study is part of a larger project named *Segurança Alimentar e Nutricional no Ambiente Escolar (Food and Nutritional Security in the School Environment)*, approved by the Research Ethics Committee of the Health Sciences Department at Universidade Federal do Paraná (UFPR), under opinion no. 316,185. This is a population-based cross-sectional study carried out between February and September 2014 with children enrolled in CMEIs from the city of Guaratuba, Paraná. In 2014, the municipality had five CMEIs, totaling 275 children aged 6 to 23 months. Among them, 19 were not allowed to participate in the study, and 43 did not bring the record of meals had at home. The final population amounted to 213 children aged 6 to 23 months.

Guaratuba is on the coast of Paraná, located 120 km from the state capital, Curitiba. Fishing, tourism, and agriculture are its main economic activities. In 2014, its estimated population was approximately 34 thousand inhabitants, of whom 1,798 were under the age of three. Currently, the estimated population is 37 thousand inhabitants, considered the second most populous city on the coast of Paraná.

The research team was trained to standardize the correct data collection procedures concerning questionnaire administration and food consumption. After signing the informed consent form (ICF), parents and guardians answered a structured and previously tested questionnaire aimed at obtaining information on socioeconomic and demographic conditions.

Food consumption data were collected from food records (weighed and estimated). At all meals, the research team directly weighed the foods offered in the CMEIs to every child included in this study. Food was weighed in a Plena^®^ portable digital scale, with 5 kg capacity and 1 g accuracy; as for liquids, the instrument used was a 10-mL graduated cylinder with 250 mL capacity. The children’s meals were identified, and the food served; repetitions and leftovers were weighed and recorded in specific forms. The amount of food consumed by each child was calculated by adding the foods served and the repetitions and subtracting the leftovers.

The estimated food record was filled by parents or guardians to obtain information about meals held at home on a weekend day, specifically Sunday. This day was chosen to facilitate parents’ participation. The form had illustrations of the type and size of utensils to assist them in detailing homemade measurements and portion sizes. In addition, the research team resolved doubts and inconsistencies when parents or guardians returned the food record.

Thus, food consumption data were obtained from all children on two non-consecutive days — a weekday including only the meals offered at the CMEI and a weekend day at home. The reported foods were distributed into seven food groups from the minimum dietary diversity (MDD) indicator, proposed by the World Health Organization (WHO):^12^

- Grains, roots, and tubers.
- Legumes and nuts.
- Milk and dairy products.
- Flesh foods.
- Eggs.
- Vitamin A-rich fruits and vegetables.
- Other fruits and vegetables.

The remaining foods were classified into six other groups adapted from the indicator proposed by the Food and Agriculture Organization of the United Nations (FAO):^13^

- Oils and fats.
- Savory and fried snacks.
- Sweets.
- Sugar-sweetened beverages.
- Condiments and seasonings.
- Other beverages.

The food groups consumed received a score of 1, and the non-consumed ones, a score of 0, resulting in a total ranging from 0 to 7. The MDD indicator was calculated based on the proportion of children aged 6 to 23 months who consumed foods from four or more food groups out of the seven proposed by the WHO.^12^

Odds ratios and their respective 95% confidence intervals (95%CI) were obtained for the following covariates in each food group: child’s age, maternal age, maternal ethnicity, maternal schooling, maternal occupation, number of children, number of household residents, child’s caregiver, household income, and being a Bolsa Família (Brazilian welfare program) recipient. Covariates with p<0.020 in the bivariate analysis (not adjusted for the effect of other covariates) were considered in the multivariate analysis (adjusted). Non-significant covariates were excluded from the model using the backward strategy. However, we chose to keep covariates with p<0.20 in the final model, even though the final conclusions were based on a 5% significance level. The intra-individual correlation, resulting from repeated measures for each child, was incorporated into the analysis by adjusting the models via the quasi-likelihood method and generalized estimating equations. Robust standard errors were calculated to overcome possible model specification problems. All analyses were performed in the statistical software R, version 4.0.2, and the geepack package.^14^

## RESULTS

Out of the 255 children who participated in the study, only those aged 6 to 23 months with a food record describing one day at CMEI and one weekend day at home were included. Thus, 213 children participated in this research. Table 1 shows the socioeconomic and demographic characteristics of the children and their mothers.

**Table 1.** Socioeconomic e demographic characteristics of children aged 6 to 23 months. Guaratuba, Paraná (n=213).

	n	%
Sex (n=213)		
Male	121	56.8
Female	92	43.2
Age in months (n=213)		
6 to 11	52	24.4
12 to 23	161	75.6
Maternal age (n=211)		
<20 years	31	14.7
≥20 years	180	85.3
Maternal schooling (n=211)		
<8 years of study	40	19.0
≥8 years of study	171	81.0
Maternal ethnicity (n=213)		
White	113	53.1
Multiracial/black	100	46.9
Maternal occupation (n=213)	163	76.5
Number of children (n=213)		
1 child	76	35.7
≥2 children	137	64.3
Household residents (n=213)		
1 to 4	137	64.3
≥5	76	35.7
*Per capita* income* (n=192)		
<½ minimum wage	90	46.9
≥½ minimum wage	102	53.1
Bolsa-Família** (n=209)	96	45.9

*Per capita household income according to the Brazilian minimum wage in 2014 (R$ 724.00/US$ 321.80); ** Bolsa-Família: Brazilian welfare program. CMEI: Centro Municipal de Educação Infantil (Municipal Nursery School).

**Figure 1.**
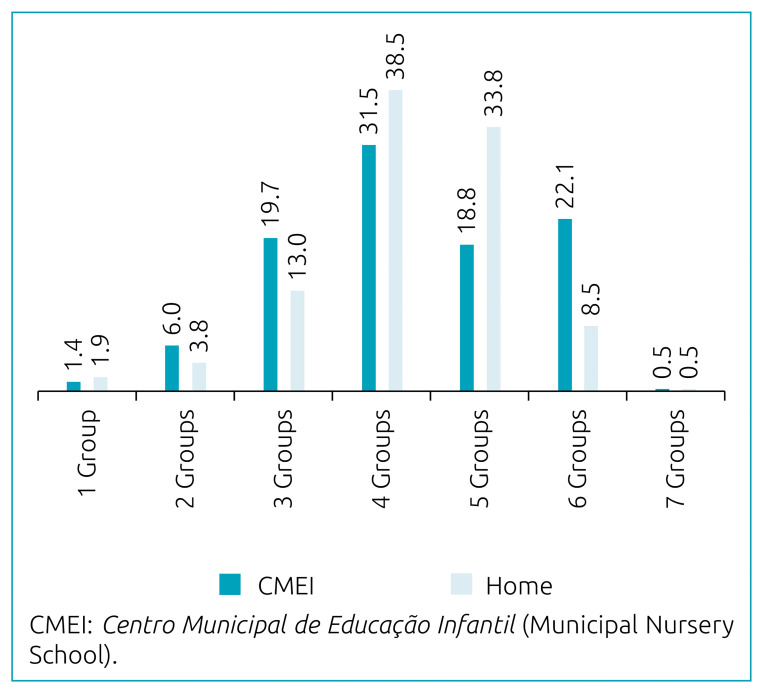
Percentage of children aged 6 to 23 months according to the number of food groups consumed in the school and home environments. Guaratuba, Paraná (n=213).

The prevalence of MDD at home was 81.2%, and the average number of food groups consumed was 4.2±1.0. We could not evaluate the prevalence of MDD in CMEIs because it represents only part of the children’s daily consumption. However, the average number of groups consumed was 4.2±1.2. Figure 1 presents the number of food groups consumed in the school and home environments.

The grains, roots, and tubers group was the most consumed in CMEIs (100.0%) and at home (99.1%), while the egg group was the least consumed, with a prevalence of 6.6 and 2.8%, respectively. The milk and dairy products group was the second most consumed at home (92.0%), a position held by the flesh foods group (94.8%) at CMEIs. Vitamin A-rich fruits and vegetables were consumed by 40.4% of children in CMEIs and 26.8% of children at home. More than 60% of children consumed sweets and sugar-sweetened beverages at home (Table 2).

**Table 2.** Prevalence of consumption of food groups in day care centers and at home among children aged 6 to 23 months (n=213).

	Food groups	CMEI n (%)	Home n (%)
MDD	Grains, roots, and tubers	213 (100)	211 (99.1)
	Legumes and nuts	132 (62.0)	106 (49.8)
	Milk and dairy products	102 (47.9)	196 (92.0)
	Flesh foods	202 (94.8)	179 (84.0)
	Eggs	14 (6.6)	6 (2.8)
	Vitamin A-rich fruits and vegetables	86 (40.4)	57 (26.8)
	Other fruits and vegetables	159 (74.6)	152 (71.4)
Other	Oils and fats	39 (18.3)	17 (8.0)
	Savory and fried snacks	11 (5.2)	53 (24.9)
	Sweets	67 (31.5)	129 (60.6)
	Sugar-sweetened beverages	83 (39.0)	131 (61.5)
	Condiments, sauces, and seasonings	118 (55.4)	24 (11.3)
	Other beverages	–	18 (8.5)

CMEI: *Centro Municipal de Educação Infantil* (Municipal Nursery School); MDD: minimum dietary diversity.

Table 3 describes the prevalence of consumption of food groups according to age group. Children aged 12 to 23 months consumed more legumes and nuts, milk and dairy products, flesh foods, and eggs both in CMEIs and at their homes. Regarding the group of vitamin A-rich fruits and vegetables, consumption was higher among children aged 6 to 11 months.

**Table 3.** Prevalence of consumption of food groups in day care centers and at home according to age group (n=213).

	Food groups	CMEI		Home	
		6 to 11 months (n=52) n (%)	12 to 23 months (n=161) n (%)	6 to 11 months (n=52) n (%)	12 to 23 months (n=161) n (%)
MDD	Grains, roots, and tubers	52 (100)	161 (100.0)	51 (98.1)	160 (99.4)
	Legumes and nuts	19 (36.5)	117 (72.7)	19 (36.5)	87 (54.0)
	Milk and dairy products	9 (17.3)	93 (57.8)	41 (78.8)	155 (96.3)
	Flesh foods	48 (92.3)	154 (95.7)	39 (75.0)	140 (87.0)
	Eggs	1 (1.9)	13 (8.1)	1 (1.9)	5 (3.1)
	Vitamin A-rich fruits and vegetables	22 (42.3)	64 (39.8)	14 (26.9)	43 (26.7)
	Other fruits and vegetables	46 (88.5)	113 (70.2)	33 (63.5)	119 (73.9)
Other	Oils and fats	3 (5.8)	36 (22.4)	1 (1.9)	16 (9.9)
	Savory and fried snacks	1 (1.9)	10 (6.2)	5 (9.6)	48 (29.8)
	Sweets	14 (26.9)	53 (32.9)	30 (57.7)	99 (61.5)
	Sugar-sweetened beverages	20 (38.5)	63 (39.1)	16 (30.8)	115 (71.4)
	Condiments, sauces, and seasonings	16 (30.8)	102 (63.4)	4 (7.7)	20 (12.4)
	Other beverages	–	–	6 (11.5)	12 (7.5)

CMEI: *Centro Municipal de Educação Infantil* (Municipal Nursery School); MDD: minimum dietary diversity.

This study identified 77 foods in the school environment, of which the ten most consumed were: soup, infant formula, rice, beans, tomato sauce, reconstituted powdered milk, infant cereals, corn starch, coffee with sugar, and beef. At home, 187 foods were reported, of which the ten most consumed were: cow’s milk, infant cereals, rice, beans, cookies, banana, sweetened juice drink, soup, French bread, and breast milk.

Table 4 presents the bivariate and multivariate analyses of the association of food group consumption with socioeconomic and demographic factors. Due to result homogeneity, the variables could not be associated with the consumption of the grains, roots, and tubers group and the egg group. The bivariate analysis related the higher consumption of legumes and nuts to children aged 12 to 23 months (p=0.030), as well as being a Bolsa Família recipient (p=0.015). Lower intake of legumes and nuts was associated with higher household income (p=0.011). Children aged 12 to 23 months were more likely to consume milk and dairy products (p<0.001), as well as flesh foods (p=0.044). The lower intake of vitamin A-rich fruits and vegetables was related to being a Bolsa Família recipient (p=0.027). At the same time, the higher consumption of other fruits and vegetables showed an association with maternal age ≥20 years (p=0.009).

**Table 4.** Association of consumption of food groups from the minimum dietary diversity indicator with socioeconomic and demographic factors.

	Categories	COR (95%CI)	p-value	AOR (95%CI)	p-value
Legumes and nuts group					
Child’s age	6–11 months	1	0.003	1	0.193
	12–23 months	2.04 (1.72–3.88)		1.57 (0.79–3.12)	
Bolsa-Família^#^	No	1	0.015		
	Yes	1.97 (1.13–3.43)			
Income*	<1/2 minimum wage	1	0.011	1	0.019
	≥1/2 minimum wage	0.47 (0.26–0.84)		0.49 (0.27–0.89)	
Milk and dairy products group					
Child’s age	6–11 months	1	<0.001	1	0.002
	12–23 months	6.93 (2.41–19.85)		5.58 (1.89–16.45)	
Bolsa-Família^#^	No	1	0.091	1	0.200
	Yes	2.73 (0.85–8.77)		2.18 (0.65–7.24)	
Income*	<1/2 minimum wage	1	0.113		
	≥1/2 minimum wage	0.38 (0.11–1.25)			
Flesh foods group					
Child’s age	6–11 months	1	0.044	1	0.044
	12–23 months	2.22 (1.02–4.83)		2.22 (1.02–4.83)	
Vitamin A-rich fruits and vegetables group					
Maternal age	<20 years	1	0.064	1	0.107
	≥20 years	2.81 (0.94–8.44)		2.49 (0.82–7.56)	
Maternal schooling	<8 years	1	0.137		
	≥8 years	1.94 (0.80–4.69)			
Bolsa-Família^#^	No	1	0.027	1	0.042
	Yes	0.48 (0.25–0.92)		0.51 (0.27–0.97)	
Other fruits and vegetables group					
Child’s age	6–11 months	1	0.149		
	12–23 months	1.63 (0.83–3.17)			
Maternal age	<20 years	1	0.009	1	0.009
	≥20 years	2.81 (1.28–6.14)		2.81 (1.28–6.14)	
Number of children	1 child	1	0.050		
	≥2 children	1.83 (1.00–3.37)			
Ethnicity	Multiracial/black	1	0.186		
	White	1.49 (0.82–2.71)			

COR: crude odds ratio; 95%CI: 95% confidence interval; AOR: adjusted odds ratio; ^#^Bolsa-Família: Brazilian welfare program; *per capita household income according to the Brazilian minimum wage in 2014 (R$ 724.00/US$ 321.80). The only results presented are those corresponding to covariates with p<0.20 in the bivariate analysis.

Table 4 presents the multivariate analysis. After adjustments for covariates, the higher consumption of the legumes and nuts group remained associated with children of families whose *per capita* income was ≥1/2 minimum wage (p=0.019). The consumption of milk and dairy products (p=0.002) and flesh foods (p=0.044) was related to children aged 12 to 23 months. Bolsa Família recipients remained associated with a lower intake of vitamin A-rich fruits and vegetables. Lastly, the consumption of other fruits and vegetables continued higher among children with mothers aged 20 years or older (p=0.009).

## DISCUSSION

The prevalence of MDD in the home environment was 81.3%, and the average number of food groups consumed was 4.2±1.0. In CMEIs, the average number of groups consumed was 4.2±1.2. Although the school environment is only partly responsible for the children’s daily food consumption, its contribution to dietary diversity was high, emphasizing the importance of food in schools. In this scenario, the National School Feeding Program (*Programa Nacional de Alimentação Escolar* — PNAE) plays a key role in forming healthy eating habits by offering a variety of foods and meeting the nutritional needs of children during their stay in day care centers.^15^

Among the seven groups described in the MDD indicator,^10^ all but milk and dairy products were more consumed in CMEIs than at home. This fact corroborates the findings of Carvalho et al.,^6^ who demonstrated the relevance of school feeding programs for complementing the meals consumed at home, contributing to nutritional adequacy.

A systematic review^16^ assessed the global prevalence of consumption of the seven food groups from the MDD indicator among children aged 6 to 23 months, based on 53 articles on food consumption in African and Asian countries. The grains, roots, and tubers group had the highest prevalence (85.3%), followed by milk and dairy products (40.8%), vitamin A-rich fruits and vegetables (34.1%), other fruits and vegetables (23%), legumes and nuts (20.6%), eggs (11.4%), and flesh foods (5.5%). The results of the present study showed similar consumption of grains, roots, and tubers and of vitamin A-rich fruits and vegetables. However, they differed as to legumes and flesh foods, which were consumed by most children in this study. This difference may be associated with the fact that the Brazilian diet consists essentially of rice, beans, and beef or chicken.^17^

Milk and dairy products represented the second most consumed group at home among children aged 6 to 11 months (78.8%). In this group, cow’s milk was the most consumed food. The offer of cow’s milk with infant cereal is very common in this age group and is associated, among other factors, with its easy preparation. Children’s consumption of milk and dairy products was also evidenced by Moraes et al.,^18^ who revealed a consumption prevalence of 83.8% in the second semester of life. Southern Brazil has one of the highest milk production and processing rates in the country, which might have contributed to the higher prevalence of milk consumption by children living in this region.^19^

Some governments and scientific societies do not recommend offering cow’s milk to children under one year,^3,20,21^ given its high content of protein, sodium, calcium, and phosphorus, low content of essential fatty acids, vitamin C, D, and E deficiency, low availability of iron and zinc, in addition to its casein-to-whey protein ratio, which compromises infant digestion and nutrient absorption.^3,22^

This study identified a lower intake of vitamin A-rich fruits and vegetables (26.8%) at home compared to CMEIs (40.4%), suggesting the influence of the family context and the school environment on children’s eating habits.^23^ Inadequate consumption of vitamin A-rich foods is common in childhood.^6^ Preliminary results from the National Study on Children’s Diet and Nutrition (*Estudo Nacional de Alimentação e Nutrição Infantil* — ENANI)^24^ indicated an 8.9% prevalence of hypovitaminosis A in Southern Brazil. Vitamin A deficiency can lead to physical and mental impairment and increased infant mortality.^25^

The consumption of UPFs at home, such as sweets (60.6%) and sugar-sweetened beverages (61.5%), was considered high among the children of this study. Morning cereals, cookies, and sweetened juicy drinks were among the most consumed foods. Other studies with Brazilian children also identified the early introduction of UPFs.^10,26,27^ Giesta et al.^10^ pointed out that, among 300 children studied, 79% had received UPFs before the age of two. Relvas et al.^26^ found that 43.1% of children aged 6 to 12 months had consumed UPFs. Lopes et al.^27^ reported consumption of morning cereals (74%), as well as *petit-suisse* cheese and sweetened yogurt (46%), among children aged 6 to 12 months. These data are alarming since the consumption of these foods by small children is associated with higher risks of developing dyslipidemia, overweight, obesity, and other cardiovascular diseases in the long term.^28^ In addition, it can create dependence and reduce the consumption of fresh and minimally processed foods.^4^

In the national context, no study has evaluated the consumption of food groups from the MDD indicator and its association with socioeconomic and demographic factors. This research revealed that the age group 12 to 23 months was associated with a higher likelihood of consuming milk and dairy products, as well as flesh foods. Neves and Madruga^29^ identified that children started consuming flesh foods later than fruits and vegetables. The *Guia alimentar para crianças brasileiras menores de 2 anos (Dietary guidelines for Brazilian children under 2 years of age)*,^4^ from the Ministry of Health, recommends offering a serving of flesh foods or eggs in the main meals after the sixth month of age, as they have protein, iron, zinc, and vitamin B12, important nutrients for the child’s proper growth and development.

A systematic review^30^ evaluating complementary feeding and its determinants found that children’s consumption of healthy foods was associated with higher household income and older maternal age. This review also identified a positive association between older maternal age and the intake of fruits and vegetables. However, children of families with higher incomes were less likely to consume legumes and nuts.

The food record can be considered a very accurate method of consumption assessment when based on direct food weighing. Nonetheless, the filling made at home may present some limitations, such as food under-reporting or the omission of foods consumed. It also requires the cooperation and literacy of parents and guardians. In addition, the collection on a weekend day, specifically Sunday, might not represent the usual home consumption, differently from the foods offered during the week in CMEIs.

This study detected a higher consumption of grains, roots, and tubers, milk and dairy products, sweets, and sugar-sweetened beverages in the home context. In CMEIs, the most consumed groups were: flesh foods and fruits and vegetables. The increased consumption of healthy foods in the school environment points to the need to promote a healthy diet among families in order to form healthy eating habits since childhood.
